# Prestroke Metformin Use on the 1-Year Prognosis of Intracerebral Hemorrhage Patients with Type 2 Diabetes

**DOI:** 10.1155/2021/2027359

**Published:** 2021-09-15

**Authors:** Wen-Jun Tu, Qingjia Zeng, Kai Wang, Yu Wang, Bao-Liang Sun, Xianwei Zeng, Qiang Liu

**Affiliations:** ^1^Institute of Radiation Medicine, Chinese Academy of Medical Sciences & Peking Union Medical College, Tianjin, China; ^2^Department of Neurosurgery, Beijing Tiantan Hospital, Capital Medical University, Beijing, China; ^3^School of Healthcare Science, Faculty of Science and Engineering, Manchester Metropolitan University, Manchester M156GX, UK; ^4^Department of Neurology, Second Affiliated Hospital of Xuzhou Medical University, Xuzhou, China; ^5^Department of Endocrinology, Affiliated Hospital of Xuzhou Medical University, Xuzhou, China; ^6^Shandong First Medical University & Shandong Academy of Medical Sciences, Jinan, China; ^7^Rehabilitation Hospital of the National Research Center for Rehabilitation Technical Aids, Beijing, China; ^8^Department of Neurosurgery, Shandong University Qilu Hospital, Jinan, China; ^9^People's Hospital of Ningjin County, Dezhou, China

## Abstract

**Background:**

Although recent studies have focused on the use of metformin in treating ischemic stroke, there is little literature to support whether it can treat intracerebral hemorrhage (ICH). Therefore, this study is aimed at evaluating the possible effects of prestroke metformin (MET) on ICH patients with type 2 diabetes.

**Methods:**

From January 2010 to December 2019, all first-ever ICH patients with type 2 diabetes from our hospitals were included. All discharged patients would receive a one-time follow-up at 1 year after admission. Death, disability, and recurrence events were recorded.

**Results:**

We included 730 patients for analysis (the median age: 65 [IQR, 56-72] years and 57.7% was men). Of those patients, 281 (38.5%) had received MET before ICH (MET+), whereas 449 (61.5%) had not (MET−). MET (+) patients had a lower median baseline hematoma volume than did MET (-) patients (9.6 ml [IQR, 5.3-22.4 ml] vs. 14.7 ml [IQR, 7.9-28.6 ml]; *P* < 0.001). The inhospital mortality events were not significantly reduced in the MET (+) group compared with the MET (-) group (6.4% vs 8.9%, respectively; absolute difference, −2.5% [95% CI, −3.9% to −0.7%]; OR, 0.70 [95% CI, 0.39 to 1.27]; *P* = 0.22). The 1-year mortality events were not significantly reduced in the MET (+) group compared with the MET (-) group (14.1% vs 17.4%, respectively; absolute difference, −3.3% [95% CI, −5.1% to −1.8%]; OR, 0.73 [95% CI, 0.47 to 1.14]; *P* = 0.16). The 1-year disability events were not significantly reduced in the MET (+) group compared with the MET (-) group (28.4% vs 34.1%, respectively; absolute difference, −5.7% [95% CI, −8.2% to −3.3%]; OR, 0.77 [95% CI, 0.52 to 1.13]; *P* = 0.18). Finally, the recurrence rates in those two groups were not significantly different (MET [+] vs. MET [-]: 6.4% vs. 5.9%; absolute difference, 0.5% [95% CI, 0.2% to 1.3%]; OR, 1.08 [95% CI, 0.51 to 2.28]; *P* = 0.84).

**Conclusions:**

Pre-ICH metformin use was not associated with inhospital mortality and 1-year prognosis in diabetic ICH patients.

## 1. Background

Metformin is the first-line drug for the treatment of type 2 diabetes mellitus [1]. It could prevent against diabetes complications [2]. Recent studies have established that metformin possesses antioxidant effects [3]. Metformin therapy could reduce oxidative stress levels in patients with type 2 diabetes [4, 5]. Metformin treatment in type 2 diabetic patients could activate oxidative stress together with the antioxidant system [6].

In addition to treating diabetes, metformin also plays an essential role in the treatment of other diseases, such as anticancer [7], antiaging [8, 9], treatment of gestational hypertension [10] and hypertension [11], and weight loss [12]. In addition, metformin use could reduce the risk of hypertension [13] and stroke [14]. The neuroprotection role of metformin in cerebral ischemia had been suggested [3, 15]. Previous studies had shown that metformin treatment had a better functional outcome in patients with diabetes and ischemic stroke [16, 17]. However, metformin therapy might also have no effect or even worsen recovery following cerebral I/R injury [18]. Another study showed that metformin use was associated with a high risk of stroke in hemodialysis patients with type 2 diabetes [19].

Furthermore, the associations between metformin treatment and prognosis in intracerebral hemorrhage (ICH) patients with diabetes have not been discussed. ICH was associated with higher mortality and poorer neurologic outcomes than ischemic stroke [20]. Qi et al. [21] suggested that metformin was a potential clinical treatment for ICH patients. It is a meaningful topic to study the use of metformin and the prognosis of ICH patients with diabetes. Therefore, this study is aimed at evaluating the possible effects of metformin on ICH patients with type 2 diabetes.

## 2. Patients and Methods

From January 2010 to December 2019, all first-ever ICH [ICD61] patients from our hospitals were screened. Patients were eligible for inclusion if they were admitted to the hospitals with a stroke defined according to the World Health Organization criteria [22]. Patients with advanced tumors, infratentorial or traumatic hemorrhage, age < 18 years, pregnancy, the transformation of cerebral infarction, and hospital stay less than 24 hours were excluded. Furthermore, ICH patients with type 2 diabetes (T2DM) were included in this study. T2DM included self-reported diabetes and newly diagnosed diabetes during hospitalization according to the WHO diagnostic criteria (fasting plasma glucose ≥ 7.0 mmol/l or oral glucose tolerance test: two − hour plasma glucose ≥ 11.1 mmol/l) [23]. The Human Research Ethics Committee of the Shandong University Qilu Hospital checked and approved the study protocol. All enrolled patients need to sign an informed consent form before enrolment.

At admission, demographic information (age, sex, race/ethnicity, body mass index [BMI], province), comorbidities and risk factors (smoking, drinking, duration of diabetes, hypertension, dyslipidemia, atrial fibrillation, coronary heart disease, coagulopathy, hyperhomocysteinemia, a family history of stroke, and transient ischaemic attack [TIA]), prestroke (antihypertensive, antiplatelet agents, statins, insulin therapy, and metformin [MET] treatment), and acute treatment were recorded. Also, the following information also had been collected: marital status, education status, the time from symptom onset to hospital arrival, transported to hospital by emergency medical service [EMS], length of stay, hospitalization costs/patient, and payment style (public medical care and self-funded medical care). Finally, discharge information, including death, discharge against medical advice, and discharge according to medical advice, were collected.

ICH severity on admission was assessed by the Glasgow Coma Scale (GCS). According to the study protocol, MRI and/or CT was used to verify the ICH diagnosed within 24 h of hospital admission. Intraventricular hemorrhage expansion, if present, was also documented. ICH volumes were quantified using the (*a* × *b* × *c*)/2 method [24]. Systolic blood pressure and diastolic blood pressure were tested at admission. Fasting serum blood samples were collected, and serum levels of glucose, homocysteine (HCY), C-reactive protein (CRP), and blood lipids were tested in the laboratory department.

### 2.1. Follow-Up

All discharged patients would receive a one-time follow-up at 1 year after admission. Outcome assessment was performed by study staff members blinded to all clinical and laboratory variables with a structured follow-up telephone interview with the patient or the closest relative. Death (all-cause), disability, and recurrence events were recorded. Functional outcomes were assessed in the follow-up by a Modified Rankin Scale (mRS) score (range from 0 to 6) [25]. Disability events were defined as an mRS of 3 to 5 points. Stroke recurrence events were defined as suddenly deteriorated neurological function evaluated as a decreased NIHSS score of 4 or more, or a new focal neurological deficit of vascular origin that lasted for >24 hours [26].

### 2.2. Statistical Analysis

Continuous and categorical variables were presented as medians (interquartile ranges [IQR]) and frequencies (%), respectively. Mann–Whitney test (Continuous variables) and chi-square test (categorical variables) were used to compare groups.

Crude rate estimate and 95% confidence interval (CI) for mortality, disability, and stroke recurrence events were assessed. The included patients were divided into two groups: MET (+) group and MET (-) group, according to prestroke MET use (yes or no). With all comparative outcomes between those two groups, cumulative rates and absolute differences with 95% CIs were presented. Regression models were performed to compare the outcomes between MET (+) and MET (-) groups, and odds ratios (OR) with 95% CIs were presented. Multivariate regression analysis adjusted for patient characteristics, including age, sex, family history of ICH, hypertension, diabetes duration, hyperlipidemia, atrial fibrillation, smoking, drinking, prestroke therapy (including oral anticoagulants, antiplatelet agents, antihypertensive treatment, and statins), ICH volumes, and NIHSS score at admission. Finally, to study MET's ability for mortality prediction, Kaplan–Meier survival curves stratified patients by MET use or not were calculated. All statistical analyses were conducted with SAS version 9.4 and Stata version 14.1. *P* < 0.05 was the threshold for statistical significance.

## 3. Results

### 3.1. Patients

**Figure 1:**
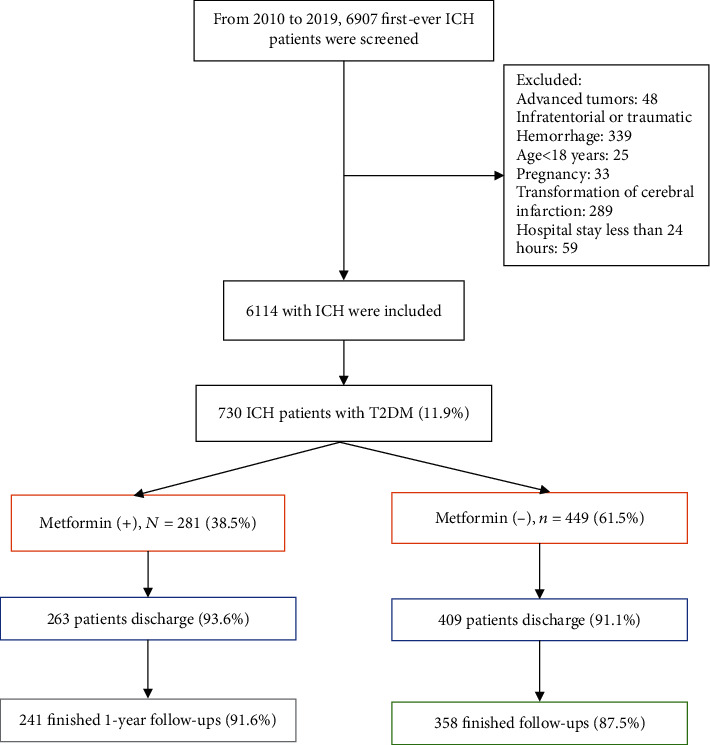
Study profile/flow sheet of the study.

From 6907, screened patients with first-ever ICH, 6114 patients were included, and 730 patients with T2DM (11.9%) were selected for analysis. Of those patients, 281 (38.5%) had received MET before ICH (MET+), whereas 449 (61.5%) had not (MET−) (Figure 1).

### 3.2. Descriptive Characteristics of Selected Patients

The median age of 730 ICH patients with T2DM was 65 (IQR, 56-72) years, and 57.7% were men. Only 2.1% of patients are younger than 40. The median duration of diabetes was 12 years (IQR, 7-18). Median systolic blood pressure was 159 mm Hg (IQR, 140-175 mm Hg), and the median diastolic blood pressure was 90 (IQR, 80-130 mm Hg). A total of 651 patients (89.2%) had a history of hypertension, 126 (17.3%) had hypercholesterolemia, 31 (4.2%) had a history of stroke, 144 (19.7%) were smokers, and 132 (18.1%) were drinkers. A total of 118 (16.2%) patients were with time from symptom onset to hospital arrival within two h, and 132 (18.1%) patients were transported to hospital by EMS. On admission, the median GCS score was 12 (IQR, 8–16) points, and the median ICH volume was 11.8 ml (IQR, 6.5-26.7). The median length of stay was 13 days (IQR, 8-21), and the median hospitalization costs 17253 CNY (IQR, 9802-39943). Demographic characteristics and clinical information of those included ICH patients were presented in Table 1.

### 3.3. Main Results

**Figure 2:**
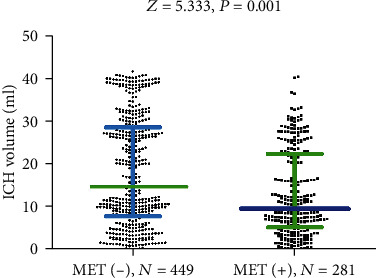
Baseline ICH volume in MET (+) patients and MET (-) patients. Mann–Whitney U Test. All data are medians and interquartile ranges (IQR), with dot plots representing all values. MET: metformin.

MET (+) patients had a lower median baseline hematoma volume than did MET (-) patients (9.6 ml [IQR, 5.3-22.4 ml] vs. 14.7 ml [IQR, 7.9-28.6 ml]; *P* < 0.001) Figure 2. MET was not associated with baseline GCS score (*P* = 0.15). In addition, MET use was not associated with age (*P* = 0.35), sex (*P* = 0.78), hospitalization stay (*P* = 0.53), and cost (*P* = 0.87).

At discharge, fifty-eight patients died, and the inhospital mortality rate was thus 7.9% (6.0-9.9%). The inhospital mortality events were not significantly reduced in the MET (+) group compared with the MET (-) group (6.4% vs 8.9%, respectively; absolute difference, −2.5% [95% CI, −3.9% to −0.7%]; OR, 0.70 [95% CI, 0.39 to 1.27]; *P* = 0.22). In addition, the discharge against medical advice events was also not significantly reduced in the MET (+) group compared with the MET (-) group (10.7% vs 11.8%, respectively; absolute difference, −1.1% [95% CI, −2.3% to −0.3%]; OR, 0.89 [95% CI, 0.56 to 1.44]; *P* = 0.64), Table 2.

In the 1-year follow-up, 73 patients lost follow-up, and 599 (90.1%) finished the follow-up. As shown in Table 2, the 1-year mortality rate was 18.0% (95% CI: 15.0%-21.1%), and the disability and recurrence rates among survivors were 31.8% (95% CI: 27.7%-35.9%) and 6.1% (95% CI: 4.0%-8.2%), respectively, Table 2.

**Figure 3:**
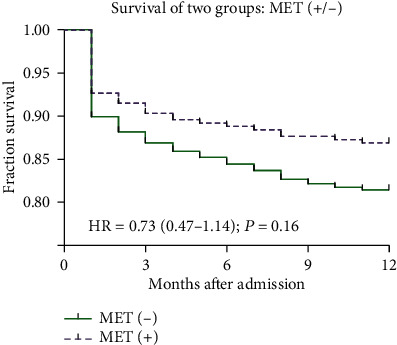
Kaplan Meier curve for all-cause mortality at 1 year for users (*n* = 241) and nonusers (*n* = 358) of MET. MET: metformin.

**Figure 4:**
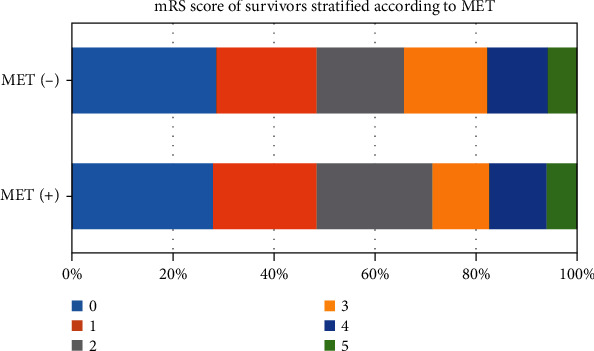
The mRS score among survivors in 1 year after admission stratified according to MET use. MET: metformin.

The 1-year mortality events were not significantly reduced in the MET (+) group compared with the MET (-) group (14.1% vs 17.4%, respectively; absolute difference, −3.3% [95% CI, −5.1% to −1.8%]; OR, 0.73 [95% CI, 0.47 to 1.14]; *P* = 0.16). The time to death was analyzed by Kaplan–Meier survival curves based on MET use. Patients in the MET (+) group did not have a minimal risk for death, in contrast with patients in the MET (-) group (*P* = 0.016) (Figure 3). The mRS score among survivors in the follow-up was shown in Figure 4. The 1-year disability events were not significantly reduced in the MET (+) group compared with the MET (-) group (28.4% vs 34.1%, respectively; absolute difference, −5.7% [95% CI, −8.2% to −3.3%]; OR, 0.77 [95% CI, 0.52 to 1.13]; *P* = 0.18). Finally, the recurrence rates in those two groups were not significantly different (MET [+] vs. MET [-]: 6.4% vs. 5.9%; absolute difference, 0.5% [95% CI, 0.2% to 1.3%]; OR, 1.08 [95% CI, 0.51 to 2.28]; *P* = 0.84).

In univariate logistic regression analysis, hematoma volume had a strong association with 1-year mortality and disability events, with the unadjusted ORs that were 1.11 (95% CI, 1.03–1.21) and 1.08 (95% CI, 1.01-1.17), respectively. After adjusting for MET use and other significant predictors, hematoma volume remained an independent outcome predictor for mortality and disability events, with the adjusted ORs that were 1.07 (95% CI, 1.01–1.15) and 1.05 (95% CI, 1.00-1.11), respectively. Furthermore, in univariate logistic regression analysis, hematoma volume did not have an association with 1-year recurrence events (*P* = 0.083).

## 4. Discussion

In this study, we assess the effect of prestroke metformin use on 1-year prognosis in ICH patients with T2DM. The data showed that pre-ICH metformin use was not associated with inhospital mortality and 1-year prognosis in diabetic ICH patients. Improving the prognosis of diabetic ICH patients by taking metformin requires further verification.

Different from our conclusion, a single-center Helsinki ICH study, including 374 ICH patients with diabetes, showed that pre-ICH metformin use was associated with improved outcomes in diabetic ICH patients [27]. It should be noted that the Helsinki ICH study was a retrospective analysis of consecutive ICH patients. In addition, the differences in acute treatment and ethnicity of the enrolled patients might have caused the inconsistency of the study results. Another study suggested that patients with ischemic stroke and diabetes on treatment with MET receiving intravenous thrombolysis had a better functional outcome at three months [17]. In addition, the use of metformin could reduce cardiovascular events in patients with T2DM [28]. Metformin treatment was associated with reduced cardiovascular risk (mortality and incidence) [29] and all-cause mortality [30] in T2DM. A meta-analysis showed that metformin reduced cardiovascular mortality, all-cause mortality, and cardiovascular events in patients with coronary artery diseases [31]. Furthermore, long-term adherence to metformin was associated with decreased risks of all-cause mortality [32]. A previous study found that metformin could be used for treating cardiovascular diseases in hypertension [33], while another study reported that metformin did not reduce blood pressure in hypertensive patients without diabetes [34]. Metformin could improve prognosis in prediabetic patients with acute myocardial infarction by reducing inflammatory tone [35]. Based on previous studies and our research conclusions, we speculated that for atherosclerosis-related diseases (such as cerebral infarction, myocardial infarction) with diabetes, MET treatment before the disease onset might improve the prognosis and in patients with cerebral hemorrhage caused by hypertension, the effect was not clear. The relationship between metformin uses, and the prognosis of hemorrhagic stroke needs further clinical research.

Previous studies showed that hematoma volume was perhaps the most important variable when determining outcomes [36–38]. In this study, we also found that hematoma volume was an outcome predictor for mortality and disability events. Furthermore, we found that MET (+) patients had a lower median baseline hematoma volume than did MET (-) patients. However, pre-ICH metformin use was not associated with 1-year prognosis in diabetic ICH patients. Thus, the relationship between metformin use, hematoma volumes, and patient prognosis could not be confirmed. Whether metformin use affects diabetic ICH patient prognosis by changing hematoma volume needs further research to verify.

Oxidative stress caused by components of the lysed erythrocytes contributes to brain injury after ICH [39], and superoxide contributes to spontaneous ICH's pathogenesis through activation of matrix metalloproteinase-9 [40]. Metformin could activate oxidative stress and the antioxidant system. Metformin promotes benefits to oxidative stress control in the muscle of hypoinsulinemic rats [41]. Metformin improves obese male fertility by alleviating oxidative stress-induced blood-testis barrier (BTB) damage [42]. Metformin inhibits oxidative stress-mediated cholesterol uptake via SREBP2 [43]. In ligature-induced periodontitis in rats, metformin use could decrease the inflammatory response, oxidative stress, and bone loss [44]. Metformin could delay vascular dysfunction in Goto–Kakizaki rats by reducing mitochondrial oxidative stress [45]. We speculated that metformin might play a role in ICH's prognosis by regulating the oxidative stress response. However, our research results did not support this conclusion, and more clinical studies need to verify the hypothesis.

Recent studies had found that metformin played roles in heart and pancreatic *β* cells [46]. The anti-inflammatory and antioxidative properties of metformin might also indirectly improve endothelial function in the cardiovascular system [47]. Bonnefont-Rousselot et al. [2] showed that metformin could directly scavenge reactive oxygen species (ROS), of which NADPH oxidase constitutes the major source. Metformin attenuated the development of atherosclerosis by reducing Drp1-mediates mitochondrial fission in an AMPK-dependent manner [48].

The neuroprotective effect of metformin had been proposed. One study suggested that metformin administration could improve neurobehavioral function following traumatic brain injury by inhibiting microglia activation-mediated inflammation via NF-*κ*B and MAPK signaling pathways [49]. Another study showed that metformin could exert a neuroprotective effect by activating the PI3K/Akt signaling pathway [50]. Also, in acute stroke patients with type 2 diabetes, metformin could improve the neurological function and oxidative stress status by the AMPK/mTOR signaling pathway and oxidative stress [51], and in acute ischemic injury, prestroke metformin treatment was neuroprotective involving AMPK reduction [52]. Metformin was a favorable target in therapeutic intervention of cerebral ischemia injury models [53].

This study has many research limitations. First, this study is a single-center and only includes Chinese. Research representativeness needs to be explained carefully. Second, observational research cannot draw causal conclusions [54]. Whether metformin use could improve diabetic ICH patients' prognosis needs further verification by randomized, double-blind controlled trials. Third, this study spans a long time, nearly ten years; during the study period, ICH patients' management plan has undergone significant changes. Lastly, newly diagnosed diabetic patients during follow-up may also use metformin, which has a confounding effect on our research. Also, detailed glucose profiles beyond admission and medical complications following admission are not collected, which could have provided insight into the potential role of metformin in ICH prognosis.

## 5. Conclusions

Pre-ICH metformin use was not associated with inhospital mortality and 1-year prognosis in diabetic ICH patients. Improving the prognosis of diabetic ICH patients by taking metformin requires further verification.

## Figures and Tables

**Table 1:** Demographic characteristics and clinical information of those included ICH patients^a^.

	ICH
*N*	730
Sex-male, *n* (%)	421 (57.7)
Age, median (IQR), years	65 (56-72)
< 40	15 (2.1)
40-60	244 (33.4)
> 60	471 (64.5)
Race-Chinese Han	708 (97.0)
Marital status-married	699 (95.8)
Education status-college and more	44 (6.0)
Patients with time from symptom onset to hospital arrival within 2 h	118 (16.2)
Transported to hospital by EMS	132 (18.1)
Smoking	144 (19.7)
Drinking	132 (18.1)
BMI, kg/m^2^	23.66 (22.01-25.53)
Systolic pressure, mmHg	159 (140-175)
Diastolic pressure, mmHg	90 (80-103)
Duration of diabetes, median (IQR), years	12 (7-18)
Comorbidities	
Hypertension	651 (89.2)
Dyslipidemia	126 (17.3)
Atrial fibrillation	29 (4.0)
Coronary heart disease	31 (4.2)
Coagulopathy	18 (2.5)
Hyperhomocysteinemia	41 (5.6)
A history of stroke	31 (4.2)
TIA	11 (1.5)
Laboratory serum testing	
GLU, mmol/l	8.35 (6.54-10.75)
TG, mmol/l	1.37 (0.97-1.96)
TC, mmol/l	4.47 (3.80-5.22)
LDL, mmol/l	2.57 (1.98-3.38)
HDL, mmol/l	1.12 (0.95-1.41)
HCY, umol/l	12.5 (10.4-16.9)
CRP, mg/l	5.8 (1.9-11.9)
Severity at admission	
GCS	12 (8-16)
mRS	3 (1-4)
ICH volume, ml	11.8 (6.5-26.7)
Prestroke treatment	
Antihypertensive treatment	505 (69.2)
Antiplatelet agents	101 (13.8)
Statins	106 (14.5)
Insulin therapy	322 (44.1)
Metformin treatment	281 (38.6)
Length of stay, days	13 (8-21)
Hospitalization costs/patient, CNY	17253 (9802-39943)
Payment style-no medical insurance	61 (8.4)

Abbreviation: ICH: intracerebral hemorrhage; GCS: Glasgow Coma Scale; mRS: Modified Rankin Scale; EMS: emergency medical service; CNY: China Yuan; IQR: interquartile ranges; TIA: transient ischemic attack. ^a^The continuous variables are presented as median with interquartile ranges (IQRs) and categorical variables as frequency and percentage (95% CI).

**Table 2:** The discharge and follow-up information of those included ICH patients stratified according to MET use^a^.

	ALL	MET use	
		MET (+)	MET (--)
Discharge, *N*	730	281	449
Died	58 (7.9 [6.0-9.9])	18 (6.4 [3.5-9.3])	40 (8.9 [6.3-11.5])
Survivors	672 (92.1 [90.1-94.0]).	263 (93.6 [90.7-96.5])	409 (91.1 [88.5-93.7])
mRS of survivors	2 (1-4)	2 (1-4)	2 (1-4)
Discharge against medical advice	83 (11.4 [9.1-13.7])	30 (10.7 [7.1-14.3])	53 (11.8 [8.8-14.8])
Follow-up at 1 year after admission	672	263	409
Lost follow-up	73 (10.9)	22 (8.4)	51 (12.5)
Finished follow-up	599 (90.1)	241 (91.6)	358 (87.5)
Died	108 (18.0 [15.0-21.1])	37 (14.1 [9.9-18.3]	71 (17.4 [13.7-21.0]
Survivors	491 (82.0)	204 (85.9)	287 (82.6)
mRS 0	139 (28.3)	57 (27.9)	82 (28.6)
mRS 1	99 (20.2)	42 (20.6)	57 (19.9)
mRS 2	97 (19.8)	47 (23.0)	50 (17.4)
mRS 3	70 (14.3)	23 (11.3)	47 (16.4)
mRS 4	58 (11.8)	23 (11.3)	35 (12.2)
mRS 5	28 (5.7)	12 (5.9)	16 (5.6)
Disability rate^a^	156 (31.8 [27.7-35.9])	58 (28.4 [22.2-34.6])	98 (34.1 [28.7-39.6]
Recurrence rate among survivors	30 (6.1 [4.0-8.2])	13 (6.4 [3.0-9.7])	17 (5.9 [3.2-8.7])

Abbreviation: mRS: Modified Rankin Scale; CI: confidence interval; MET: metformin. ^a^Disability event was defined as an mRS of 3 to 5 points.

## Data Availability

Please contact the corresponding author for data requests.

## Additional Points

*Research in context*. *What is already known about this subject?* Metformin is the first-line drug for the treatment of type 2 diabetes mellitus. Metformin use could reduce the risk of hypertension and stroke. Metformin treatment had a better functional outcome in patients with diabetes and ischemic stroke. *What is the key question?* The associations between metformin treatment and prognosis in intracerebral hemorrhage (ICH) patients with diabetes have not been discussed. *What are the new findings?* The inhospital mortality events were not significantly reduced in the MET (+) group compared with the MET (-) group (6.4% vs 8.9%, respectively; absolute difference, −2.5% [95% CI, −3.9% to −0.7%]; OR, 0.70 [95% CI, 0.39 to 1.27]; *P* = 0.22). The 1-year mortality events were not significantly reduced in the MET (+) group compared with the MET (-) group. The 1-year disability and recurrence events were not significantly reduced in the MET (+) group compared with the MET (-) group. *How might this impact on clinical practice in the foreseeable future?* Pre-ICH metformin use was not associated with inhospital mortality and 1-year prognosis in diabetic ICH patients.
